# Adipose tissue-derived stem cells in breast reconstruction: a brief review on biology and translation

**DOI:** 10.1186/s13287-020-01955-6

**Published:** 2021-01-06

**Authors:** Jun Fang, Feng Chen, Dong Liu, Feiying Gu, Yuezhen Wang

**Affiliations:** 1 Zhejiang Key Laboratory of Radiation Oncology, Hangzhou, China; 2 Department of Radiation Therapy, Zhejiang Cancer Hospital, Hangzhou, China; 3 Radiotherapy, Institute of Cancer and Basic Medicine (ICBM), Chinese Academy of Sciences, Cancer Hospital of the University of Chinese Academy of Sciences, Zhejiang Cancer Hospital, Hangzhou, China; 4 Department of Breast Tumor Surgery, Institute of Cancer Research and Basic Medical Sciences of Chinese Academy of Sciences, Cancer Hospital of University of Chinese Academy of Sciences, Zhejiang Cancer Hospital, Hangzhou, China

**Keywords:** Adipose tissue-derived stem cells, Breast reconstruction, Biological characteristics, Translational significance

## Abstract

Recent developments in adipose-derived stromal/stem cell (ADSC) biology provide new hopes for tissue engineering and regeneration medicine. Due to their pluripotent activity, paracrine activity, and immunomodulatory function, ADSCs have been widely administrated and exhibited significant therapeutic effects in the treatment for autoimmune disorders, neurodegenerative diseases, and ischemic conditions both in animals and human clinical trials. Cell-assisted lipotransfer (CAL) based on ADSCs has emerged as a promising cell therapy technology and significantly improved the fat graft retention. Initially applied for cosmetic breast and facial enhancement, CAL has found a potential use for breast reconstruction in breast cancer patients. However, more challenges emerge related to CAL including lack of a standardized surgical procedure, the controversy in the effectiveness of CAL, and the potential oncogenic risk of ADSCs in cancer patients. In this review, we summarized the latest research and intended to give an outline involving the biological characteristics of ADSCs as well as the preclinical and clinical application of ADSCs.

## Introduction

Fat grafting is widely used in plastic surgery for various diseases in recent decades, due to its advantages of biocompatible, easy access, cost-effective, less complications, and less damage to the donor sites [1]. However, with the development of liposuction and various survival fat volume prediction tools, although autologous fat grafting technology has been gradually improved over a century time, the loss rate of fat grafts remains the greatest problem which ranges from 20 to 90% at 1 year after surgery [2] and the survival volume could not be accurately predicted [3, 4].

In 1999, Pittenger et al. proposed for the first time that human bone marrow stem cells (BM-MSCs) were stable and pluripotent in vitro that could be induced to differentiate exclusively into adipocytic, chondrocytic, or osteocytic lineages without evident necrosis or apoptosis, indicating that MSCs were potentially novel therapy for trauma repair or diseased tissue through cell planting technology [5]. Since then, cell therapy based on MSCs has developed and found an important role in the field of tissue engineering and regeneration medicine [6]. Zuk et al. demonstrated for the first time that adipose tissue was also abundant in multipotent stem cells, namely adipose-derived stromal/stem cells (ADSCs) [7]. ADSCs are demonstrated to have similar self-renewal, unlimited proliferation capacity, and pro-angiogenic capacity and can increase the concentration of pro-angiogenic factors, promote epithelial cell differentiation and neovascularization, and ultimately improve fat grafts survival and plastic effects [7–10]. ADSCs can also regulate local and systemic biological responses through both cell-to-cell communication and paracrine manner, including regulating immune responses, inhibiting apoptosis, promoting angiogenesis, mediating inflammatory responses, and matrix remodeling [11–13]. Due to these practical advantages, ADSC-based cell therapy has been widely applied for wound healing [14–16], soft tissue regeneration [17, 18], and bone generation [19]. Therefore, along with the mature liposuction technologies to obtain ADSCs with less invasive, low cost, and higher ADSCs yield than bone marrow [20], ADSCs seem more suitable for fat grafts enhancement. Thus, surgeons mixed an additional percentage of isolated and in vitro cultured ADSCs into lipoaspirate to obtain a novel fat graft, which was called cell-assisted lipotransfer (CAL) [21]. Both preclinical and clinical studies suggested that ADSC-based CAL could improve the survival of fat grafts and has been well used especially in breast augmentation and facial lipoatrophy [22]. Nevertheless, due to the complication and economic limitations of isolation and expansion procedures of ADSCs, surgeons prefer to applying stromal vascular fraction (SVF) instead to deliver fresh ADSCs for CAL in clinical trials rather than ADSCs. Briefly, SVF is a mixture of cell populations that derives from lipoaspirate component by removing mature adipocytes after centrifugation. SVF contains abundant ADSCs (about 10%), vascular endothelial cells as well as their precursors with different differentiation degrees [23]. SVF also contains certain percentage of macrophages, smooth muscle cells, lymphocytes, pericytes, and fibroblasts [23], which provides an abundantly cellular and molecular microenvironment for regulating ADSCs under clinical conditions. Studies have shown that SVF-based CAL could improve fat graft survival [21, 24, 25]. SVF technology is also widely used in clinical trials for wound healing, joint conditions, and urogenital and cardiovascular diseases [26], exhibiting pro-angiogenesis and neovascularization effect. Nevertheless, the relatively low number of published clinical studies, lack of standard protocol, and financial loss hinder the application of ADSC- or SVF-based cell therapy in clinical work.

Breast cancer patients are a special population different from breast augmentation and trauma patients. Although the safety of fat injection for breast reconstruction has been broadly confirmed by a large number of clinical trials [27], in vitro and in vivo studies in animal models have found that both adipocytes and ADSCs can affect the breast cancer microenvironment and promote breast cancer growth and metastasis [28–32]. This proposes concerns regarding the use of fat ADSC-enriched CAL therapy in breast reconstruction after oncology surgery. In recent years, preclinical studies and clinical trials have been made to explore the safety and effectiveness of ADSCs in breast reconstruction and obtained some encouraging results. A meta-analysis by Laloze et al. in 2018 found that ADSC-based CAL can significantly improve the fat survival (64% vs. 44%) in small-volume fat grafting (less than 100 ml) without increasing the risk of tumor recurrence within 1 year after surgery [33]. However, large cohorts and longer follow-up are needed to affirm the safety of CAL technology in patients with malignancy.

This review aims to provide a brief review on both the clinical and molecular evidences on the role of ADSCs in breast cancer and potential application of ADSC-based CAL in breast reconstruction after oncology surgery.

## Phenotypic characterization of ADSCs

ADSCs mostly distribute in perivascular niche [34]. Compared with bone marrow, ADSCs yield from fat tissue liposuction is 500-fold greater with a much less invasive manner [20]. Through in vitro expansion, ADSCs show a fibroblast-like morphology, similar to BM-MSC [7]. Actually, the exact phenotype of ADSCs remains unclarified because surface biomarkers differ depending on donor cites and culture passages in vivo and in vitro [23]. According to a joint statement issued by the International Federation of Fat Therapy and Science (IFATS), ADSCs retain similar phenotypic markers to BM-MSCs and are commonly positive for CD44, CD73, CD90, and CD105 and negative for CD31 and CD45 [35]. By the way, ADSCs also express moderate CD36 and CD106^−^, which can usually be distinguished from BM-MSC [35].

The expression of CD34 in ADSCs is of interest because the connection between CD34 and ADSCs functions remains to be controversial. CD34 is limited to ADSCs freshly isolated or cultured within 8–12 passages in vitro, indicating that CD34 plays as a niche-specific marker of immature cells or precursors [35, 36]. So far, CD34 has been considered to be related to the “stemness” of ADSCs. Suga et al. compared biological functions of CD34^−^ and CD34^+^ ADSCs and demonstrated that CD34^+^ correlated to greater proliferation capacity, lower differentiation potential, and higher expression of endothelial progenitor markers, whereas CD34^−^ ADSCs expressed higher markers of pericyte [37]. CD34^+^ ADSC also exhibited greater capacity in promoting breast cancer proliferation, metastasis, and angiogenesis in animal models [38]. Nevertheless, when CD34 turned negative after long-time expansion in vitro (25 passages), ADSCs still showed strong proliferative ability and multi-differentiation potential [37, 39].

## Basic functions of ADSCs

### Pluripotent differentiation ability

ADSCs represent a group of heterogeneous cells exhibiting similar pluripotential ability to MSCs in vitro and in vivo and can differentiate into endodermal-, mesoderm-, and ectodermal-derived cells [40]. In addition to the most common adipocytic, chondrocytic, and osteoblastic lineages presented in studies, ADSC can also differentiate into epithelial cells, endothelial cells, liver cells, and nerve cells as well [35]. This potential is physically essential to support local tissue-specific precursors in faced with damage for tissue regeneration. Moreover, ADSCs exhibit several unique characteristics compared with BM-MSCs. ADSCs exhibit a longer survival time, stronger proliferative capacity, shorter doubling time, and later in vitro senescence [41]. Even cultured for 3 months, ADSCs still have the potential to differentiate into adipocytes [42].

### Paracrine and angiogenic activity

ADSC-derived secretome has intrigued increasingly attention in tissue regeneration area. ADSCs can secrete a variety of extracellular vesicles (EVs), exosomes, and soluble factors [43]. An extensive range of chemokines, cytokines, growth factors, mRNAs, and micro-RNAs are released by ADSCs that participate in angiogenesis, lymphangiogenesis, immune modulation, and reducing fibrogenesis [22, 43]. These anti-apoptotic factors and pro-angiogenic factors can affect activities of the nervous system, immune system, heart, muscles, and even ordinary somatic cells through endocrine and paracrine methods and have been demonstrated to play a therapeutic role in bone reconstruction, nerve protection, heart regeneration, and soft tissue regeneration [43–47]. However, the capacity of EVs to secrete growth factors and promote tissue regeneration was indicated not as strong as ADSCs [47]. ADSCs can also produce antioxidants, free radical scavengers, and heat shock proteins (HSP), which promote the repair of viable cells by removing harmful substances from the injured tissue and accelerate wound healing [48, 49]. In addition, when stimulated by growth factors or inflammatory molecules, expression profiles of ADSCs may vary accordingly. For example, ADSCs stimulated by basic fibroblast growth factor (bFGF) or epidermal growth factor (EGF) released significantly more hepatocyte growth factor (HGF), which further leads to more synthesis of ascorbic acid [50].

Due to the pluripotent and paracrine potential, studies have applied ADSCs for cell therapy and tissue engineering in ischemia diseases [51]. In a myocardial ischemia mouse model, a small portion of ADSCs could differentiate into endothelial cells and vascular smooth muscle cells [52], as well as cardiomyocytes [53]. More importantly, high expression of VEGF was detected in the marginal area of the heart infarct zone when treated by ADSCs, thereby improving myocardial function and avoiding adverse ventricular remodeling [54]. In a cerebral ischemia rat model, infusion of ADSCs conditioned medium (CM) into the lateral ventricle or vein might reduce the infarct volume and nerve cell apoptosis, promote the proliferation of vascular endothelial cells, and increase the density of microvessels [55, 56]. For the avascular necrosis or ulcer wounds caused by diabetes, ADSCs implantation could also obtain a significant therapeutic effect [57]. In addition, researchers found that external stimulation such as FGF-2 [58], hypoxia [59], or recombinant adeno-associated virus (rAAV) serotype 2 encoding human VEGF165 [60] can further enhance the paracrine effect of ADSCs and thus promote angiogenesis.

### Immunomodulatory properties

The immunosuppressive function of BM-MSCs has been widely reported [61]. BM-MSCs can suppress both innate and adaptive immune systems by direct cell-to-cell communication and paracrine cytokines [13]. They could suppress the activation of cytotoxic T cells and differentiation of T helper (Th) cells through the production of immunosuppressive molecules such as indoleamine 2,3-dioxygenase (IDO), transforming growth factor beta (TGF-β), heme oxygenase 1 (HO1), and nitric oxide (NO) [62, 63]. PGE2 was reported in vitro to be involved in the inhibition of allogeneic lymphocyte reaction [64]. Patricia et al. identified IL-1RA as a key factor involved in mediating the process of MSCs inhibiting B lymphocytes differentiation and macrophages differentiation into the M2 subgroup [65]. In addition to inhibiting the proliferation and differentiation of cytotoxic cells such as T cells, NK cells [66], B cells [67], and dendrite cells (DC) [68], MSCs can also induce the generation and expansion of Foxp3^+^ regulatory T cells [69]. One study indicated that CD5^+^ regulatory B cells could also be promoted by MSCs and ameliorated refractory chronic GvHD [70].

As a perfect substitute for BM-MSCs, ADSCs even have a stronger capacity than BM-MSCs to secrete immunosuppressive factors such as IL-6 and TGF-β [71]. With the increase of ADSCs passage number, the expression levels of hematopoietic-related factors and histocompatible locus antigen-DR (HLA-DR) on ADSCs gradually decreased and ADSCs beyond passage P1 failed to elicit T cells activation and suppressed MLR response [72]. Both in vitro and in vivo results indicated allogeneic ADSCs presented immune tolerance and could not stimulate lymphocyte proliferation, which allows the possibility of ADSCs xenotransplantation [73]. Preclinical studies and clinical trials have shown that ADSCs have a good therapeutic effect in various autoimmune diseases [74–76].

### Origin of ADSCs

Fat tissue is distributed throughout the human body at subcutaneous and visceral depots, such as abdominal, thigh, omentum, and pericardium. Due to the abundance of fat at subcutaneous sites, the CAL surgeries are always based on ADSCs isolated from abdominal subcutaneous fat or breast. Recently, the comparative studies begun to explore the differences in ADSCs populations isolated from various anatomical locations. They indicated that ADSCs were influenced significantly by the microenvironment of a specific tissue source in cell phenotype, cell proliferation ability, differentiation ability, and apoptosis susceptibility [77–80]. For example, although ADSCs derived from breast fat and abdominal subcutaneous fat are similar in cell phenotype and genetic characteristics, ADSCs from breast fat have higher self-renewal capacity and are more likely to differentiate into osteoblasts, whereas ADSCs from subcutaneous fat are more predisposed to the adipogenic lineage, suggesting that the latter one seems more suitable for fat grafting [81]. Valerio et al. compared the ADSCs from subcutaneous, omental, and intrathoracic (pericardium, pericardial fat, thymic remnant) fat tissue depots and concluded that ADSCs isolated from intrathoracic depots had the longest doubling time, omentum ADSCs displayed the highest level of osteogenic markers while lowest adipogenic differentiation capacity, and the subcutaneous fat-derived ADSCs demonstrated enhanced adipogenic differentiation capacity [82]. The high potential of subcutaneous fat-derived ADSCs to differentiate into adipocytes was consistent with the previous studies [79, 83]. Schipper et al. especially compared the ADSCs from different parts of subcutaneous (upper arm, medial thigh, trochanteric, and both superficial and deep abdominal) fat depots and found that variations in proliferation capacity and apoptotic susceptibility were dependent on donor age and depot [78]. ADSCs from the superficial abdominal depot displayed the lowest apoptosis level, and those derived from young patients presented the highest proliferation capacity [78]. Jin et al. further demonstrated that ADSCs derived from younger populations not only possessed higher proliferative capacity, migratory capacity, and T cell suppressive ability, but also more predisposed to osteoblast differentiation [84]. Therefore, referring to breast reconstruction, it seems more appropriate to utilize ADSCs from young donors and abdominal subcutaneous fat tissue.

### ADSCs and breast cancer

Although the role of ADSCs in the development and metastasis of breast cancer has not been fully clarified, it has been concerned for a long time that ADSCs may increase the risk of breast cancer recurrence after CAL-based breast reconstruction.

ADSCs primarily exist in fat tissue, as well as the breast cancer microenvironment. Besides the resident ADSCs and those inoculated by white adipose tissue grafting, ADSCs could also be recruited from the blood to tumor center. The investigators label adipose tissue with GFP and found that ADSCs injected into the circulation or transplanted with adipose tissue could be recruited to breast cancer and further differentiated into vascular endothelial cells, fibroblasts, and pericytes [85, 86]. Moreover, ADSCs could selectively home to and engraft into tumor stroma to promote tumor growth and invasion, while filtering organs such as the lung, liver, and spleen were rarely ADSC-enriched, suggesting that tumor was the prerequisite of ADSCs homing and vascular engraftment [85].

Razmkhah et al. compared the secretion cytokines between ADSCs from breast cancer adjacent adipose and those from healthy breast tissues and found that the latter secreted higher levels of VEGF, IL-8, HGF, and IGF-1, suggesting tumor-related ADSCs potentially had greater ability to promote angiogenesis and tumor growth [87]. However, no matter ADSCs were isolated from breast cancer adjacent adipose, benign breast tumor adjacent adipose, or normal breast adipose, all cells were able to promote the proliferation of MCF-7 breast cancer cells in vitro, and cancer-associated ADSCs seemed to exhibit more significant effect than ADSCs from normal fat tissue [88]. Multiple signaling pathways such as HGF/c-Met, miR20b, and IGF1/IGFBP2 are involved in the process of ADSCs promoting tumor cell proliferation [89–91]. ADSCs can also upregulate the epithelial-mesenchymal transition markers of breast cancer cells, such as fibronectin, alpha smooth muscle actin, and vimentin, and promote the ability of tumor metastasis and invasion [89, 92, 93]. Through paracrine manner by secreting FGF-2 and activating extracellular signal-regulated kinase (ERK), ADSCs could drive tumor cell proliferation at the chemo-residual triple-negative breast cancer [94]. ADSCs might even fuse with tumor cells to form hybrid cells which possessed stronger proliferation and metastasis capabilities [95].

In contrast, some other studies indicated contradictory results. Donnenberg et al. showed that ADSCs enhanced the growth of active but not resting breast tumor cells [96]. Kucerova et al. directly co-cultured SKBR3 breast cancer cells with ADSCs and their CM and found that although ADSCs and CM could promote the expression of EMT markers and cell migration ability, both of them unexpectedly inhibited the proliferation of SKBR3 breast cancer cells and enhanced the sensitivity to chemotherapy [97]. In mouse models, MSCs isolated from adipose and umbilical cord blood even reduced breast cancer lung metastases and induced tumor cell necrosis [98]. A recent study compared the effects of ADSCs from different breast adipose sources (invasive breast cancer, BRCA-mutated invasive breast cancer, ductal carcinoma in situ, healthy controls) on multiple breast cancer cell lines and found that ADSCs from invasive breast cancer patients displayed a significant enhancement on the proliferation and metastasis of JIMT in vivo and in vitro, which was HER2-enriched and anti-HER2 resistant, but exhibited a weak promotion effect on T47D and no effect on MDA231 [99].

CM and EVs of ADSCs have been encountered similar discrepancy. Sakurai et al. treated multiple breast cancer cell lines with ADSC-derived CM which result in enhanced cell proliferation and migration capacity and further identified the possible involvement of calcium-binding protein S100A7 in this process [100]. On the contrary, Trivanović et al. found that CM of ADSCs inhibited the growth of MCF-7 cell line in vitro [101]. Wu et al. further discovered that CM could activate the DNA damage response pathway ATM-Chk2 cascades in MCF-7, MDA-MB231, and MDA-MB468 breast cancer cell lines, thereby inhibiting cell proliferation and inducing apoptosis [102]. As we have reviewed above, EVs could carry ADSC-specific proteins and RNAs to target cells and affect them. ADSC-derived EVs delivered abundant pro-angiogenic factors, which contributed to promoting microvascular endothelial cells to form vessel-like structure in vitro and more neovessels within the fat graft in vivo [103, 104]. Lin et al. also confirmed that ADSC-derived EVs could promote the proliferation and metastasis of MCF-7 breast cancer cells by activating the Wnt/β-catenin signaling pathway [105]. However, EVs are heterogeneous, and if EVs highly express tumor suppressor molecules such as MiR379, they turn into tumor growth inhibitors and might be a potential therapeutic tool [106]. In general, the influence of ADSCs on the proliferation and metastasis of breast cancer is still inconclusive which might partly owe to the heterogeneity of ADSCs cell population, state of breast cancer cells, and the complicated microenvironment of cancer; therefore, further research is needed.

ADSCs can also be educated by tumor cells. When co-cultured with breast cancer cells, ADSCs would differentiate into cancer-associated fibroblast-like myofibroblastic phenotype, namely CAFs, express α-SMA and tenascin-C, and promote angiogenesis and cell invasion in breast cancer [99, 107]. It has been demonstrated that water-soluble molecules secreted by breast cancer cells, such as α-SMA, TGF-β, IL-8, and MMP-3, could not only recruit ADSCs from adjacent tissues, but also promote the proliferation, proangiogenic factor secretion, and myofibroblastic differentiation of ADSCs and inhibit adipogenic differentiation of ADSCs, leading to extracellular matrix deposition and contraction [30]. Some other permanent changes in transcriptome would occur as well in educated ADSCs, including upregulation of brain-derived neurotrophic factor, neurogenic locus notch homolog protein 1, and cytoskeletal vimentin and downregulation of growth differentiation factor 15, insulin-like growth factor 1, matrix metallopeptidase-2, and platelet-derived growth factor receptor-βand transforming growth factor-β3 [99]. Even mature adipocytes are transformed into cancer-associated adipocytes when co-cultured with breast cancer cells, exhibiting an altered phenotype characterized by delipidation, decreased adipocyte markers, and overexpression of proinflammatory cytokines expressing higher levels of pro-inflammatory factors such as IL-6. IL-1β in turn induced breast cancer cells into a more aggressive behavior phenotype [108]. More signaling pathways related to ADSC activation and re-programming remain to be discovered as future therapeutic targets.

## Cell-assisted lipotransfer (CAL)

Both autologous transplantation and breast implants are widely used for breast reconstruction. Autologous flap transplantation is the gold standard and provides a quite esthetic, natural, and biocompatible alternative [109], while it is accompanied with trauma at the donor site, wound cracking, flap necrosis and loss, the risk of abdominal wall hernia, high operating difficulty, and long hospital stay [110]. Breast prosthesis has become the most commonly used reconstruction technique due to its advantages of short operation time, donor area morbidity-free, and enhanced recovery after surgery, but it also has some defects, such as secondary replacement surgery, repeat prosthesis replacement, prosthesis-related soft tissue infection, capsular contracture, prosthesis displacement, rupture of the prosthesis, and breast implant-associated anaplastic large cell lymphoma [111]. In contrast, with the development of liposuction applied for obtaining fat tissue, autologous fat graft (AFG) has become the most promising breast reconstruction technology due to less damage to donor sites. Even for patients after breast-conserving surgery, the esthetic appearance of the breast could be improved by small volume AFG [112].

The most important challenge for AFG is to raise the fat survival to get a better appearance, especially for patients who had mastectomy surgery. Currently, the survival rate of AFG is averagely 50%, and necrosis of fat cells occurs inevitably leading to cysts and reduction of fat volume. Therefore, multiple AFG surgeries are always needed to achieve a satisfactory appearance. Compared with adipocytes, ADSCs are more resistant to ischemia and hypoxia, thus contributing to adipose tissue repair under ischemic conditions [113]. Research indicated that although liposuction reduced the damage to the donor area, ADSCs were 50% less than the adipose bulk due to mechanical damage [24]. Suga et al. proposed that 100 ml of adipose tissue contained about 10^8^ stem cells, while processing 100 ml of fat particles through the Celution® system could only obtain 2.5–4.0 × 10^7^ stem cells [113]. Therefore, deficiency of multipotent ADSCs might partly explain the predisposition of fat graft to necrosis. In order to improve the fat survival rate, researchers added additional b-FGF, VEGF, platelet-rich plasma, and ADSCs to the fat graft and indeed achieved encouraging results [114–117]. As reviewed above, ADSCs possess a series of features, including being able to differentiate into a variety of cell types including adipocytes; secreting abundant growth factors such as VEGF, HGF, FGF-2, and IGF-1 in a paracrine manner; and modulating immune responses resulting in immune tolerance, which makes ADSCs a perfect alternative for adipose tissue engineering. ADSC-based soft tissue engineering has developed rapidly in the past decades, and ADSCs gradually become the main source of stem cells for adipose engineering including breast reconstruction [118].

### Application of CAL in animal models

In 2006, Yoshimura et al. firstly proposed the concept of CAL. ASC-based CAL commonly mix ASC-enriched SVF or isolated and in vitro expanded ADSCs with the fat graft to make an ADSC-enriched graft. They mixed SVF in human aspirated fat and transplant subcutaneously into immunodeficient mice, ultimately promoting microvessels formation at the graft and improving the fat survival (35% larger than the non-CAL group on average) [24]. It has been well known that ADSCs has lower oxygen consumption and better tolerance than adipose cells when faced with hypoxic environment during fat transplantation. Meanwhile, ADSCs could be induced by αvβ3 integrin and serpin family E member-1 and migrate to perivascular niche, where they proliferated, differentiated into vascular endothelial cells, adipocytes, and granulosa cells, thereby promoted angiogenesis and reduced tissue necrosis [117, 119, 120]. Furthermore, high fraction of ADSCs can also recruit stem cells from other sites, especially the bone marrow, into adipose tissue, and further increase VEGF levels and fat transplant survival [121]. In summary, for most animal models, including models with immunodeficiency and normal immune, the use of ADSC-based CAL can significantly improve the survival of fat grafts [122–124].

In order to improve the fat survival further, research adding VEGF, CXCR4, bFGR, or PRP into ADSCs for CAL indicated improvement in the efficiency of CAL [125–128]. Due to the extensive expression of estrogen receptor in adipose tissue [129], Sai et al. pretreated ADSCs with estrogen and observed increased secretion of VEGF, greater adipogenic differentiation and proliferation capacity of ADSCs, and higher survival of fat graft in the mouse model [130]. However, a lot of details remain clarified and additional studies are needed on the mechanisms and treatment modification of CAL. For example, Ko et al. applied human passaged ADSCs for CAL at mice model and found that the fat graft volume of the CAL group was larger and central fat necrosis was lower than control at week 4 after surgery while no difference between the two groups at 15 weeks, suggesting that the effects of ADSCs on the duration of fat graft need further studies to explore [131]. Li et al. explored the effects of PRP and different densities (10^5^/ml, 10^6^/ml, 10^7^/ml) of ADSCs on fat graft and suggested that 10^5^/ml ADSCs plus PRP group exhibited the highest fat survival and the most significant angiogenesis rather than 10^7^/ml [117]. The researchers believed that it took a certain period of time for ADSCs to differentiate into adipocytes and a larger density of ADSCs might not form normal adipose tissue but promoted formation of dense connective tissue at the early stages, which was not in favor of the migration of ADSCs to the perivascular niche or the formation of capillary beds and thus hindered the differentiation of ADSCs [117], while these hypotheses need future studies to clarify the interaction between ADSCs and microenvironment (Tables 1 and 2).

**Table 1:** Studies in favor of that ADSCs may increase the risk of breast cancer

Reference	Year	ADSCs origin	ADSCs surface marker	Results
Razmkhah et al. [103]	2010	Human breast (normal versus cancer-affected)	CD44^+^ CD105^+^ CD166 ^+^ CD14^−^ CD34^−^ CD45^−^	Cancer-affected ADSCs expressed higher levels of VEGF, IL-8, HGF, and IGF-1
Yan et al. [104]	2012	Human breast (normal versus cancer-affected)	CD29^+^CD73^+^CD90^+^CD105^+^ CD166^+^ CD31^−^CD144^−^CD14^−^CD45^−^ HLA-DR^−^	ADSCs promoted the proliferation of MCF-7 via EGF/EGFR/Akt (cancer-affected > normal)
Devarajan et al. [92]	2012	Human whole fat	–	ADSCs promoted the proliferation and EMT markers expression of multiple breast cancer cell lines
Orecchioni et al. [105]	2013	Human lipoaspirates	CD13^+^CD34^+^CD140b^+^ CD31^−^CD45^−^	ADSCs promoted the proliferation of multiple breast cancer cell lines (MDA-MB-436, HCC1937, and ZR75–1) and EMT markers expression in ZR75–1
Lin et al. [106]	2013	Human lipoaspirates	–	ADSC-derived exosomes promoted the proliferation of MCF-7 cells via activating Wnt signaling
Eterno et al. [107]	2014	Human lipoaspirates and breast whole fat (normal versus cancer-affected)	CD44^+^CD90^+^CD117^+^ CD133^+^ CD34^low^CD45^−^	ADSCs are not tumorigenic; ADSCs stimulated the proliferation, migration and tumorigenic behavior of MDA-MB-231 but not MCF-7 via HGF/c-Met
Trivanović et al. [108]	2014	Human breast (normal vs. cancer-affected); abdominal	CD44^+^CD73^+^CD90^+^CD105^+^ CD11a^−^CD33^−^CD45^−^CD235a^−^	ADSCs from all sources exhibited similar character and enhanced the proliferation of MCF-7 via direct co-culture
Sakurai et al. [109]	2017	Human subcutaneous fat	–	ADSC-derived CM promoted the proliferation of multiple breast cell lines
Melzer et al. [110]	2018	Umbilical cord explant cultures	–	The hybrid of ADSCs with MDA-MB-231 cells developed elevated metastatic capacities.
Xu et al. [111]	2019	Mice inguinal fat tissues	–	ADSCs stimulated the migration and invasion of 4T1 cells via c-Kit/MAPK-p38/E2F1 signaling
Lyes et al. [112]	2019	Zenbio (ASC-F)	–	ADSCs CM increased proliferation of the chemo-residual TNBC cells relying on FGF2
Fajka-Boja et al. [113]	2020	Mice visceral fat tissues	–	ADSCs promoted 4T1 cells growth and metastasis via IGF/IGFBP

**Table 2:** Studies contradicted to the promotion effect of ADSCs on breast cancer

Reference	Year	ADSCs origin	ADSCs surface marker	Results
Sun et al. [117]	2009	Human breast fat	–	ADSCs reduced lung metastasis and inhibited the growth of human breast cancer cells by inducing apoptosis in mice model
Zimmerlin et al. [115]	2011	Human Subcutaneous adipose tissue	–	ADSCs enhance the growth of active metastatic pleural effusion cells, but not resting tumor cells
Kucerova et al. [116]	2013	Human lipoaspirates	CD44^+^CD73^+^CD90^+^CD105^+^ CD14^−^CD34^−^CD45^−^	ADSCs inhibited the proliferation of SKBR3 cells via SDF-1α/CXCR4 and increased chemosensitivity in a paracrine manner
Trivanović et al. [108]	2014	Human breast (normal versus cancer-affected) and abdominal	CD44^+^CD73^+^CD90^+^CD105^+^ CD11a^−^CD33^−^CD45^−^CD235a^−^	ADSC-derived CM showed antiproliferative activity
Wu et al. [119]	2019	Human lipoaspirates	–	ADSC-derived CM reduced cell viability by triggering apoptosis in MCF-7, MDA-MB231, and MDA-MB468 cells via ATM-Chk2-dependent DNA damage response
Plava et al. [118]	2020	Breast adipose tissue of healthy donors, adipose tissue adjacent to pre-malignant lesions or malignant lesions or malignant lesions with BRCA mutation	CD90^+^ CD105^+^ CD73^+^ CD14- CD20^−^ CD34^−^ CD45^−^	Cancer-related ADSCs displayed a significant enhancement on the proliferation and metastasis of JIMT but exhibited a weak promotion effect on T47D and no effect on MDA231

### Application of CAL in human

Two years after introduction of CAL, Yoshimura et al. firstly applied CAL in humans and affirmed that the SVF-based CAL achieved performance in 40 patients for breast augmentation [21]. The soft and natural-appearing augmentation did not change significantly after 2 months after surgery, and only 4 patients developed cysts or microcalcifications, which supported the effectiveness and safety of CAL. Since then, numerous clinical trials on CAL emerge. Despite encouraging results in animal models, the therapeutic effect of CAL in human breast reconstruction and plastic surgery is still controversial. Kølle et al. mixed ADSCs (2 × 10^7^ cells/ml) with 30 ml of adipose tissue in vitro and planted them under the skin of the upper limbs of healthy subjects [132]. Results indicated that fat survival of the ADSC-enriched group was significantly higher than the control group (80.9% vs. 16.3%, *P* < 0.0001) at 121 days after surgery, and no serious adverse events occurred. The study conducted by Gentile et al. also supported the advantages of SVF-based CAL that SVF enhanced survival of autologous fat grafts significantly (63% vs. 39%, *P* < 0.0001) at 1 year after surgery [133]. In contrast, results from Wang et al. [134] and Peltoniem et al. [135] were completely opposite that SVF did not improve fat survival in 10 and 12 patients undergoing breast augmentation, respectively, and the fat reabsorption rates were still around 50% at 6 months after surgery. A lot of factors contribute to the variations of results from different studies, including age of fat donor [136], SVF or ADSCs density, ADSCs isolation technique, fat transplantation surgery process, surgeon skills, and blood supply of the recipient site. It demonstrated that about 90% of the ADSCs remained viable at 1 h after isolation, while only 6% remained active after 6–8 h [137]. Therefore, the isolation of ADSCs from adipose tissue should be as soon as possible and careful operation is very important to improve the therapeutic effect of CAL. Similar to the results of ADSCs in animal models, high-density SVF was proved to be better than low-density for CAL and when the number of SVF cells was 10 times higher, the fat survival volume was increased by 25% (75% vs. 50%, *P* < 0.01) at 1.5 years after surgery, suggesting that increasing SVF density is also possible strategy to improve fat survival [138].

The risk of developing a tumor is rather low in the patients for breast augmentation, and cosmetic appearance is the most important issue. While for breast cancer after oncologic surgery, in addition to cosmetic satisfaction, the most important concern is the oncogenic risk of ADSCs. Breast microenvironment after breast cancer surgery is completely different from a normal breast. As mentioned above, ADSCs have functions including immune regulation, immunosuppression, cellular homing, pro-angiogenesis, and anti-apoptosis, which endow them not only the application in tissue engineering, but also the possibility to induce breast cancer development and progression. Therefore, although the role of ADSCs in breast cancer has not been fully elucidated, the application of CAL in breast cancer patients for reconstruction must be cautious and follow-up after surgery should be close. However, the microenvironment of human adipose tissue is much more complicated than conditions of in vitro experiments or animal models; results of preclinical experimental studies cannot be directly adopted for clinical trials. For example, in recent years, adipocytes have been proved to promote the conversion of androgen to estrogen via expressing aromatase [139] and alter the tissue environment in a paracrine manner [140], thus promoting the progress of breast cancer [141]. However, in clinical practice, the safety of fat grafts has been widely recognized. Studies indicated that fat transplantation does not increase the local tumor recurrence risk in breast cancer patients [142–145]. Meantime, results of two studies are noteworthy. One is a case-control study conducted by *Kronowitz* et al., in which the local recurrence risk among the overall lipofilling population was equal to the control group, while subgroup analysis indicated increased risk (1.4% vs. 0.5%, *P* = 0.038) among the lipofilling population who received endocrine therapy [146]. This difference might partly attribute to the fact that patients who received lipofilling tend to be elderly, lower tumor stages, and receive more endocrine therapy, which is in line with the real-world situation. Whether there is cross-talk between endocrine therapy and effects of ADSCs remains elucidate. The other study was a case-control study among intraepithelial neoplasia patients [147]. Petit et al. found that fat grafting after surgery for intraepithelial neoplasia increased the 5-year local tumor recurrence risk (18% vs. 3%, *P* = 0.02), especially among the subgroups of patients older than 50-year-old patients, with high histological grade, with higher ki67, and receiving breast-conserving surgery. The tumor recurrence rate in this study was significantly higher than other studies, which might attribute to the short time (most within 3 years) of fat grafting from the radical tumor resection surgery, because most breast cancer recurrence appeared in the first several years after surgery [148]. Although these two studies are retrospective, they still suggested a valuable concern about the safety of fat transplantation. In addition, the follow-up time in most studies is less than 8 years, while breast cancer has a persistent risk of recurrence for 20 years after surgery. Therefore, it is too early to conclude that fat transplantation is absolutely safe.

Up to now, the clinical practice of CAL in the breast reconstruction is very limited, and most studies are retrospective analysis or case-control. Prospective research is rare, so there is no high-level evidence to support the application. In a preclinical study, Lee et al. injected fat and SVF subcutaneously in a breast cancer xenograft mouse model and found that SVF-based CAL significantly increased the fat survival volume and did not promote the growth of nearby breast tumor [149]. Mazur and Calabrese and others conducted retrospective clinical studies and found that SVF-based autologous fat transplantation did not increase the tumor recurrence rate within 3 and 5 years, affirming the safety of CAL [150, 151]. The RESTORE-2 trial was the first prospective trial to evaluate the effect of the CAL technique for breast-conserving breast cancer [152]. ADSC enrichment of fat grafts significantly improved graft survival and residual volume (over 80%). Although 35.7% (24/67) of the patients underwent the second operation at 6 months after the primary surgery, 85.1% (57/67) patients were satisfied with the cosmetic appearance after 12 months. Meanwhile, no local tumor recurrence or serious adverse events occurred after CAL. The main adverse event was cyst at the injection site. However, since the follow-up time of this study is only 12 months, the safety of CAL also remains explored in the future.

## Summary

**Fig. 1:**
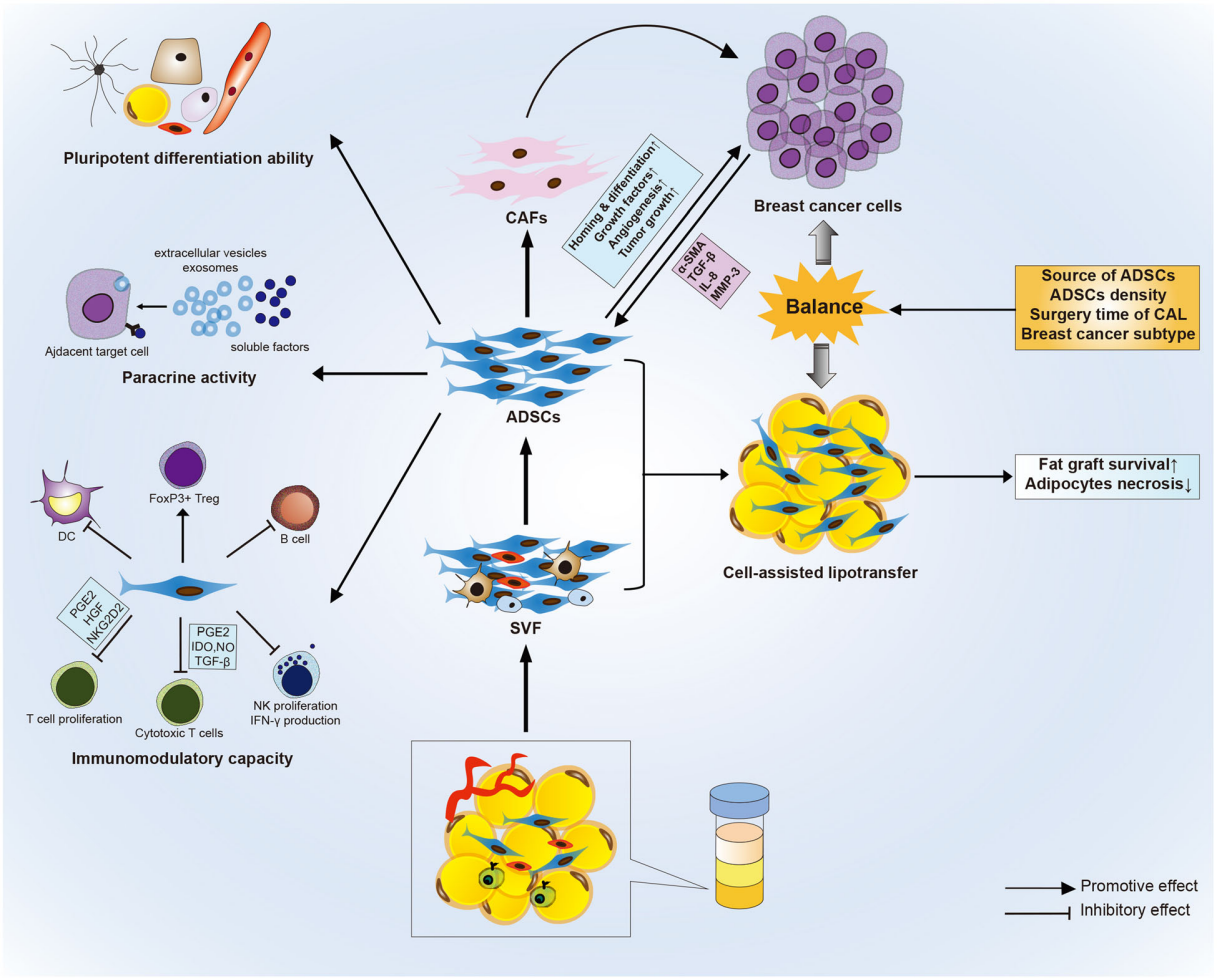
Capacities of adipose tissue-derived stem cells and their potential for cell-assisted lipotransfer (CAL)

This review identifies a solid foundation and a promising trend for ADSC-based CAL technology in breast reconstruction. The multiple capacities including pluripotent activity, paracrine activity, immunomodulatory function, and pro-angiogenesis entitle ADSCs to be a perfect product for regenerative medicine (Fig. 1). Though the evidence levels of clinical practice up to now are limited, most trials favor the advantage of CAL therapy in breast reconstruction without increasing oncogenic risk, encouraging more large cohorts to apply this technology and provide higher-level evidence. A notable breakthrough for MSC therapy recently was the authorization of allogeneic ADSCs by the European Medicines Agency to treat complex perianal fistulas in Crohn’s disease in 2018 [153], intriguing a lot of ongoing trials applying ADSC-based cell therapy for soft-tissue reconstruction, neurodegenerative diseases, and ischemic conditions.

Some areas to be improved include the timing of ADSCs transplantation, standardized surgical procedures, comparison of clinical benefits of ADSCs and SVF, ex vivo expansion skills of ADSCs, and the relationship between ADSCs and various adjuvant treatments. It is expected that more investigators will propose well-designed prospective clinical studies to further solve these problems. Pre-clinical studies tracking the complexity of ADSCs in tumor microenvironment will also deliver useful information to optimize CAL technology.

## Data Availability

Not applicable.

## Abbreviations

ADSCs: Adipose-derived stromal/stem cells
SVF: Stromal vascular fraction
CAL: Cell-assisted lipotransfer
BM-MSCs: Bone marrow stem cells
IFATS: International Federation of Fat Therapy and Science
IHC: Immunohistochemistry
TAFs: Tumor-associated fibroblasts
CM: Conditioned medium
EVs: Extracellular vesicles
HSP: Heat shock proteins
bFGF: Basic fibroblast growth factor
EGF: Epidermal growth factor
HGF: Hepatocyte growth factor
TNF-α: Tumor necrosis factor-α
IGF-1: Insulin-like growth factor-1
VEGF: Vascular endothelial growth factor
MAPK: Mitogen-activated protein kinase
LPS: Lipopolysaccharide
GM-CSF: Granulocyte-macrophage colony stimulating factor
IDO: Indoleamine 2,3-dioxygenase
TGF-β: Transforming growth factor beta
HO1: Heme oxygenase 1
NO: Nitric oxide
iNOS: Inducible nitric oxide synthase
PGE2: Prostaglandin E2
MLR: Mixed lymphocyte reaction
DC: Dendritic cells
HLA-DR: Histocompatible Locus antigen-DR
hrVEGF: Human recombinant vascular endothelial growth factor
ECs: Endothelial cells
VSMCs: Vascular smooth muscle cells
GRO: Growth-regulated oncogene
EAN-78: Experimental autoimmune neuritis-78
MCP-1: Monocyte chemoattractant protein-1
rAAV: Recombinant adeno-associated virus
ERK: Extracellular signal-regulated kinase
AFG: Autologous fat graft
